# Internal consistency of demographic assumptions in the shared socioeconomic pathways

**DOI:** 10.1007/s11111-014-0206-3

**Published:** 2014-02-12

**Authors:** Leiwen Jiang

**Affiliations:** National Center for Atmospheric Research, Boulder, CO USA

**Keywords:** Socioeconomic scenarios, Demographic assumptions, Internal consistency, Climate change

## Abstract

A new set of alternative socioeconomic scenarios for climate change researches—the shared socioeconomic pathways (SSPs)—includes for the first time a more comprehensive set of demographic conditions on population, urbanization, and education as the central scenario elements, along with other aspects of society, in order to facilitate better analyses of challenges to climate change mitigation and adaptation. However, it also raises a new question about the internal consistency of assumptions on different demographic and economic trends under each SSP. This paper examines whether the interactions between the demographic and economic factors implied by the assumptions in the SSP projections are consistent with the research literature, and whether they are consistently represented in the projection results. Our analysis shows that the interactions implied by the demographic assumptions in the SSPs are generally consistent with findings from the literature, and the majority of the assumed relationships are also evident in the projected trends. It also reveals some inconsistency issues, resulting mainly from the use of inconsistent definitions of regions and limitations in our understanding of future changes in the patterns of interactions at different stages of socioeconomic development. Finally, we offer recommendations on how to improve demographic assumptions in the extended SSPs, and how to use the projections of SSP central elements in climate change research.

## Introduction

A new set of alternative socioeconomic scenarios for climate change research—the shared socioeconomic pathways (SSPs)—includes for the first time a comprehensive set of demographic factors on population, urbanization, and education, along with other aspects of socioeconomic conditions, such as gross domestic product (GDP), technology, and governance. It presents a major step forward as compared to the earlier IPCC socioeconomic scenarios (Nakicenovic et al. 2000) that only include projections of total population size, considered mainly as a scaling factor for assessing challenges to climate change mitigation. In the process of developing the SSPs, the climate change research communities have identified the most important and quantifiable demographic (population, education, urbanization) and economic (GDP) factors as central scenario elements critical for meeting both mitigation and adaption challenges (Schweizer and O’Neill 2013) and have projected changes in these four factors for the twenty-first century under each SSP.

**Fig. 1:**
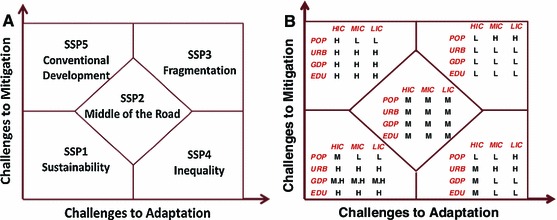
Qualitative narratives (**a**, on the *left*) and the assumptions (**b**, on the *right*) on central elements for countries by income under SSPs. *Note* the central elements are *POP* population, *URB* urbanization, GDP, *EDU* education, the income groups are *HIC* high income, *MIC* medium income, *LIC* low income; assumptions on future changes are *H* high scenario, *M.H* medium high scenario, *M* medium scenario, *L* low scenario

The SSPs start from qualitative narratives, describing five different development pathways (Fig. 1 Panel A), under which the world faces different levels of challenges to adaptation (along the horizontal axis) and to mitigation (along the vertical axis). Sustainable development is the main organizing principle in SSP1, in which technology advances toward an environmental-friendly process, inequality is lessened, and a small but highly educated and urbanized population is well positioned both to mitigate and to adapt. This is in sharp contrast to SSP3, a fragmented world with large challenges to both mitigation and adaptation due to rapid population growth, slow economic growth, less investment in human capital, slow urbanization, and high inequality. The SPP2 reflects an intermediate case between SSP1 and SSP3. SPP4 represents a world where adaptation challenges dominate due to high inequality across and within countries, where relatively rapid technological advance leads to relatively large mitigative capacity, while economies are isolated for the benefit of elites, leaving the less educated majority of population in poorly managed municipalities with limited adaptive capacity and so highly vulnerable to climate change. The SPP5 represents a world where mitigation challenges dominate because of a focus on conventional economic growth, lacking incentives for developing alternative energy technologies to meet high energy demand, but at the same time, having slower population growth, high investment in human capital, strong institutions for managing resources and development, and so therefore less vulnerable and better able to adapt to climate impacts.

Building on the qualitative narratives several research teams have taken steps to quantitatively project the trends of these central elements under each of the SSPs using various modeling tools: population and education projections by IIASA (KC and Lutz 2012); urbanization projections by NCAR (Jiang and O’Neill 2012); and GDP projections by OECD (Dellink and Chateau 2012), IIASA (Cuaresma 2012), and PIK (Kriegler and Leimbach 2012). All the projections are based on assumptions made for countries of high-, medium-, and low-income groups by each modeling team (Dellink and Chateau 2012), based on discussions in the working group “IAM quantitative drivers and IAM scenarios for SSPs,” formed by the climate change research communities.

Panel B of Fig. 1 summarizes these assumptions on *changes* 1 in the four central elements for countries by income under each SSPs. Along the diagonal of the chart, the growth rates in urbanization, per capita GDP and education, are assumed high (medium high for GDP) under SSP1, medium under SSP2, and low under SSP3 for all countries. The assumptions on population are the same for medium- and low-income countries—low under SSP1, medium under SSP2, and high under SSP3, but different for high-income countries—medium under SSP1 and SSP2, low under SSP3. These assumptions are made with the intention that the challenges to both mitigation and adaption increase from SSP1 to SSP2, and to SSP3. On the off-diagonal matrix spaces, however, the assumptions under SSP4 and SSP5 show some combinations, which differ from those in the conventional architecture of socioeconomic scenarios, such as in the previous IPCC assessment reports. Under SSP4, low GDP and education growth but high urbanization growth are assumed for medium- and low-income countries, while medium growth in all the three elements is assumed for high-income countries; population growth is assumed high for low-income countries but low for high- and medium-income countries. Assumptions under SSP5 are similar to those under SSP1, except high population growth is assumed for the high-income group, and higher GDP growth is assumed for all countries.

On the one hand, this new set of alternative socioeconomic scenarios, and the quantitative projections of the central elements will facilitate better assessments of challenges to climate change mitigation by the integrated assessment modeling (IAM) communities and will meet the needs of the impacts, vulnerability, and adaptation (IAV) communities for analyzing climate change impacts. On the other hand, it raises a new question about the internal consistency of the assumptions used in the projections about changes in the demographic and economic trends between different SSPs and across regions within one SSP, especially given that the projections are generally conducted by different modeling teams and by using different modeling tools.

Scholars often consider two types of internal consistency tests in validating model results: (1) the internal consistency of individual projections, and (2) the internal consistency within and between groups of projections. The first type of consistency test has already been conducted by each modeling team, proving their assumptions and results to be consistent with either observed data, empirical analysis, or existing theories (Cuaresma 2012; Dellink and Chateau 2012; Jiang and O’Neill 2012; KC and Lutz 2012; Kriegler and Leimbach 2012).

In this paper, we focus on the second type of internal consistency test; this is especially important given that each modeling team makes its assumptions regarding the element (or elements) it focuses on without explicitly accounting for the effects of other elements. For instance, the impacts between population/education and urbanization are not explicitly modeled in either the population/education or urbanization projections; while the economic projections use population and education as input, the direct impact of urbanization is not considered.

Even though the assumptions in the projections of individual elements are made separately (except the population and education projections, which are made in an integrated model by IIASA), the combination of assumptions on individual elements under each SSP implies certain types of interactions between these elements. We can assign for each two elements a positive relationship when they both are concomitant under high, medium or low assumptions or a negative relationship when one of the two elements is under high and the other under low assumptions. When one of the two elements is under medium assumption and the other under either high or low assumption, their relationship can be either positive or negative. The six sets of implied interactions between each two of the four elements for countries of three income groups under five SSPs are summarized in Table 1. It suggests the implied relationships under SSP2 are all positive because all elements are assumed medium changes. Similarly, the implied interactions between education and GDP (Edu*GDP) are positive for all income groups under each SSP. The positive interactions between urbanization and GDP (Urb*GDP) and between urbanization and education (Urb*Edu) are also implied in all cases, except for medium- and low-income groups under SSP4. The implied relationships between population and each of the other three elements are more varied: for low- and medium-income groups, they are all negative under SSP1, SSP3, and SSP5, but mixed under SSP4; for high-income groups, they are all positive under SSP3 and SSP5, but positive or negative under SSP1 and SSP4.

**Table 1:** Implied interaction between assumed changes in urbanization, GDP, population, and education for countries by income under SSPs

	Urb*GDP	Urb*Pop	Urb*Edu	Pop*GDP	Pop*Edu	Edu*GDP
*SSP1*						
High income	+	+/−	+	+/−	+/−	+
Medium income	+	−	+	−	−	+
Low income	+	−	+	−	−	+
*SSP2*						
High income	+	+	+	+	+	+
Medium income	+	+	+	+	+	+
Low income	+	+	+	+	+	+
*SSP3*						
High income	+	+	+	+	+	+
Medium income	+	−	+	−	−	+
Low income	+	−	+	−	−	+
*SSP4*						
High income	+	+/−	+	+/−	+/−	+
Medium income	−	−	−	+	+	+
Low income	−	+	−	−	−	+
*SSP5*						
High income	+	+	+	+	+	+
Medium income	+	−	+	−	−	+
low Income	+	−	+	−	−	+

“Urb*GDP” refers to the relationship between urbanization and per capita GDP, and so on

“+” indicates a positive correlation, “−”indicates a negative correlation, “+/−” indicates the relationship can be either positive or negative

* GDP* gross domestic product, *SSP* shared socioeconomic pathway

Are these implied interactions in the SSP projections theoretically reasonable and empirically evident? Are they consistently reflected in the projection results? These are the two main questions this paper addresses. In the next section, we review the research literature on relationships between the demographic and economic factors based on empirical studies or economic modeling, and examine whether the implied interactions in the SSP projections are consistent with findings from the literature. We then analyze the SSP projection results, study how the projected changes in the four factors are correlated, and compare them to the implied relationships. Based on these analyses, we make recommendations in the final section on how to use the projection results of the SSP central elements, and discuss how to improve future works for developing the extended SSPs for the climate change researches at the regional and local levels.

## Comparing implied interactions and interactions suggested by existing research

We review the literature on the interactions between changes in population, urbanization, education, and economic growth based on empirical evidence or economic and demographic modeling. The review is conducted for the relationships between each pair of the four factors, considering the bidirectional causal effects. The findings on the interactions suggested by the literature are then compared with those implied by the assumptions in the SSP projections in order to test the internal consistency of those assumptions.

### Urbanization and population

#### Urbanization affects population

The majority of studies of the impacts of urbanization on population growth point to negative effects. Studies in industrialized countries find that urban areas in the early stage of urbanization play the role of a “population sink” (Dyson 2003), because higher population density is associated with higher death rates before the onset of epidemiological and demographic transitions. Infectious diseases thrived in towns, where people lived at relatively high densities and interacted at high rates, resulting in a crude death rate higher than the birth rate, under which the urban sector in the long run would not exist without rural-to-urban migration (de Vries 1990). Moreover, degrading urban environments posed health hazards to both wealthy and low-income settlements in most countries, leading to the proliferation of disease vectors (Goldstein 1990). In more recent decades, HIV infections are generally higher in urban areas than those in the rural, particularly in Sub-Saharan Africa (Caldwell et al. 1997; Douglas et al. 2001).

The negative effect of urbanization on population growth is found to be more salient in modern societies because of lower fertility. Studies in many European countries and the USA indicate substantially lower fertility rates in urban areas than that of rural since the early nineteenth century (Jaffe 1942; Merrick 2001; Rutstein 2002; Singh and Casterline 1985; UN 1987). Selective migration to urban areas also leads to lower national fertility in countries such as India (Mohan 1984), China (Guo et al. 2012; Zeng and Vaupel 1989), and the Philippines (Hiday 1978). Moreover, rural out-migration may accelerate fertility transition through changing preferences for reproduction and leads to smaller family size. While the rural-to-urban migrants of first generation may not substantially change their reproductive behavior (Hawley 1971) and present higher cumulative fertility than native urban residents (White et al. 2005), other studies found urbanization reduced fertility even within the first generation in Puerto Rico and Bangkok (Goldstein 1973), Bombay (Visaria 1969), and Ghana (White et al. 2005).

Urbanization can also positively affect population growth in recent decades due to its strong impacts on improving health (Jayasuriya and Wodon 2002). Better and more efficient health facilities and services in urban areas helps reduce mortality and morbidity, increase life expectancy, and contributes to population growth. However, this positive contribution of urbanization on population growth through reducing mortality is significantly smaller than its negative effect due to fertility reduction.

#### Population affects urbanization

Studies on the impacts of population growth on urbanization mainly suggest a positive effect. Goldstein (1990) indicates that population growth increases the density of rural settlements that are consequently reclassified as urban. The high urban growth rate in many less developed countries since the 1950s is mainly caused by rapid population growth (Mohan 1984) and has resulted in 16–48 % increases in urbanization in Latin America, Sub-Saharan Africa, East Asia, and South East Asia during 1950–2010, significantly faster than the historical trend experienced by the industrialized regions (Dyson 2003).

While high urbanization growth rates are largely driven by high population growth, a slowing down of national population growth does not necessarily cause a reduction in the urbanization growth rate (Mohan 1984). When the annual national population growth rate decreased from 3.0 to 1.5 % in the 1970s in some Latin American countries, the urbanization growth rates only declined from 6–7 % to 4–5 %. Moreover, zero population growth accompanied by technological progress and income rise can increase the urbanization growth rate. Under certain conditions, a reduction in population growth may speed up the rate of urbanization (Mohan 1984). The experience of Japan and several other modern economies shows that increasing agricultural productivity and growing wealth reduces income elasticity of demand for food and causes a shift of consumption patterns toward urban goods, which in turn results in increasing demand for urban labor and high rates of urbanization even when the population growth rate decreases.

### Urbanization and education

#### Urbanization affects education

Gary Becker (1964, 1993) and his followers argue that urbanization leads to specialization and technological innovation due to higher competition, and therefore increases returns on investment in education. Urbanization is a strong determinant of the efficiency of countries in improving education outcomes (Glaeser 1997), because in the urban areas it is typically cheaper to provide access to education services and easier to monitor performance and attract quality inputs (Hardoy et al. 2001; Jayasuriya and Wodon 2002). Empirical studies in the USA indicate that urbanization in 1930–1960 increased the average size of schools, reduced the number of school districts with small enrollment, and contributed to improving education efficiency (Havighurts 1967). However, the positive impact of urbanization on human capital accumulation only occurs above a certain threshold level of development (Bertinelli and Zou 2008). Because of the limitation in human capital and rudimentary technology, the education systems in many poor countries are not able to benefit from increase in population density (Becker 1993). Bertinelli and Zou (2008) find that an urbanization level below 40 % deters human accumulation and suggest an (inverted) U-shaped relation between urbanization and education.

Others suggest a negative effect of urbanization on education. Stamp (1968) argues that urbanization is a significant cause of education problems for both rural and urban schools in Canada, because it reduces school attendance and teacher’s salary in the rural ones, and makes it hard meeting the education needs of the rapid growing industrial cities. Rapid urbanization in Africa has also been associated with a large number of young school leavers who join the large flow of out-migration (Byerlee 1974) and degrades the education system. The study by Jayasuriya and Wodon (2002) also shows that a very high level of urbanization (above 60 %) may lead to decrease in efficiency for net primary enrollment. Therefore, urbanization is a double-edge sword (Bertinelli and Zou 2008): Too little urbanization limits accumulation of human capital, while too much or too rapid urbanization growth rate also causes difficulties in meeting education demands.

#### Education affects urbanization

Studies on the effects of education growth on urbanization are scarce. Preston (1979) indicates that rural out-migration probabilities are higher among those with higher education attainment, reflecting greater returns to urban residence for the educated than for the non-educated. The selectivity of migration for more productive workers further contributes to city growth and urban economic advantage (Greenwood 1981, 1997). Therefore, education is usually associated with more urbanization. On the other hand, improving education in rural areas may dampen the flow of individuals who move to the urban for acquiring more education.

### Urbanization and economic growth

#### Urbanization affects economic growth

Many economists argue that urbanization leads to economic success because of clustering in cities, economies of scales, knowledge spillover, efficient supply of external inputs, common pool of high skill workers, higher specialization of labor, and technological advancement (Henderson 2003; Krugman 1991; Lucas 1988). A body of historical scholarship strongly suggests that urbanization, spatial inequality, industrialization, and economic development are positively related (Bairoch 1993; Ciccone and Hall 1996; Hohenberg 2004; Hohenberg and Lees 1985; Hussain et al. 2009). Glaeser (2011) finds that the correlation coefficient between the logarithm of per capita GDP and urbanization is 0.7, that a 10 % increase in urbanization is associated with a 61 % increase in per capita GDP; he maintains that to a certain degree city growth reflects a global transition from poverty to prosperity.

The positive impact of urbanization on productivity is largely due to increasing capital mobility, which leads to lower transaction costs. However, excessive concentration in the urbanization process can also increase congestion that limits capital mobility and increases transaction costs (Baldwin and Martin 2004). Therefore, in the urbanization process, gains of agglomeration work against the costs of static congestion diseconomies; and the relative importance of the two effects changes across stages of urbanization and development (Bertinelli and Black 2004). The potential gains from agglomeration matter most at early stages of development, when transportation and communication infrastructure is scarce, the reach of capital markets is limited, and efficiency can be significantly enhanced by concentrating production in space, while the congestion diseconomies do not change with the level of development per se. When infrastructure improves and markets expand, the relative importance of congestion will increase with the level of urbanization and development (Bertinelli and Black 2004; Williamson 1965). Henderson (2003) suggests that there is an optimal level of urbanization or urban concentration in terms of maximizing productivity, which varies with the level of development and country size. More importantly, productivity growth is not strongly affected by urbanization per se; it is the form of urbanization or the degree of urban concentration that strongly affects productivity growth.

Given the variations of urbanization impacts in different stages and forms of urbanization, statistical analyses using cross-country data from various time periods with different coverage of regions and countries reveal some negative effects of urbanization on economic growth. Ades and Glaeser (1995) find that both urbanization and urban primacy have a significantly negative effect on economic growth. Brulhart and Sbergami (2009) reveal that while urbanization generally leads to high productivity, the effect is reversed when a country achieve US$ 10,000 per capita GDP; Gallup et al. (1999) indicate that while urbanization is conducive to economic growth in coastal regions, increasing urban density in the interior has the opposite effect. Bloom et al. (2008) find no evidence that urbanization levels affect economic growth rates. They argue that although there is strong evidence that workers in urban areas are individually more productive and earn more due to economies of scale and richer market structure, rapid urbanization is associated with crowding, environmental degradation, and other impediments to productivity.

#### Economic growth affects urbanization

Urbanization is often regarded as primarily an economic process (Berry 1973; Mohan 1984). Williamson (1988) points out that the rapid urban expansion since 1800 is the result of economic growth due to industrialization. According to Davis (1955), the origins of urban centers are due to improved agricultural productivity. Advances in agriculture increase the productivity of farmers and create food surplus, resulting in the influx of rural migrants to the urban areas (Todaro and Smith 2003). Lacking technical change or productivity growth in agriculture is the reason for slow urbanization in many less developed countries (Mohan 1984). The changing employment opportunities due to national economic growth and changes in industrial structure are basically the underlying explanation of urbanization (Jones 2003). The economic history of many industrial and post-industrial nations has seen a dramatic rise in urbanization as the national economies developed (Hughes and Cain 2003).

However, in recent decades, urbanization sometimes occurs in places where there is little or no economic growth. In particular, Sub-Saharan Africa urbanized rapidly despite protracted negative economic growth (Fay and Opal 1999). Urbanization independent from economic growth was also observed in Latin America in the 1920s–1930s (Davis and Casis 1946; Hoselitz 1957). In other regions, some countries have even experienced urbanization during periods of economic stagnation or decline (Oucho and Gould 1993). In most of those cases, the farmers fled impoverishment and starvation in rural areas and migrated to the city with little hope of improving their standard of living (Todaro and Smith 2003). A cross-section and time-series analysis of 35 developing nations indicates a negative relationship between urbanization and urban primacy and economic growth (Abdel-Rahman et al. 2006). For the nations tested, those years in which urban population was growing faster were the years in that their economies were growing the least.

### Population and Education

#### Population affects education

Studies aimed at quantifying the mechanisms underlying the correlations between population growth and human capital formation are limited (Rosenzweig 1988). However, there exists empirical support for either a positive or negative contribution from population growth to education achievement. Weiner (1991) finds that declining population growth triggers educational expansion, not only because a small student population makes government services less costly, but also because declining birth rates are associated with greater parental willingness to send their children to school due to their changed view of children from being considered assets to being considered liabilities. Therefore, once a country starts its demographic transition, the greater parental demand for education and lower marginal costs of education will lead to expansion of mass education. On the other hand, Beaudry and Green (2002) argue that high population growth promotes adoption of high technology and makes up for relative scarcity of physical capital, which leads to high incentive to invest in education. Statistical analysis of historical records suggests that one-third of the increase in literacy is explained by population density (Boucekkine et al. 2009).

#### Education affects population

Empirical studies reveal that educational attainment makes a positive contribution to population growth through reducing mortality, but a negative contribution through reducing fertility. KC and Lentzer (2010) indicate that in virtually all societies, better educated individuals have lower mortality rates, and their children have better chances of survival. Similar findings can be found in early studies of the USA (Antonovsky 1967; Crimmins and Saito 2001; Elo and Preston 1996; Kitagawa and Hauser 1973; Pappas et al. 1993; Rogot et al. 1992), Europe (Deboosere and Gadeyne 2009; Ezendam et al. 2008; Fawcett et al. 2005; Feldman et al. 1989; Huisman et al. 2005; Kunst et al. 2004; Mackenbach et al. 2003; Mackenbach et al. 1999; Valkonen 1989), Korea (Khang et al. 2004; Son et al. 2002), Japan (Sakata et al. 2012), China (Liang et al. 2000; Zhu and Xie 2007), Bangladesh (Hurt et al. 2004; Mostafa and Ginneken 2000), and Vietnam (Huong et al. 2006).

While the positive contribution of education to population growth through reducing mortality is more obvious before and during the early stage of demographic transition, improving educational attainment induces much stronger negative effects on population growth due to fertility reduction in the later stages. Educational differentials in fertility are among the best established and most widely studied (Caldwell 1982; Cleland and Rodriguez 1988; Cochrane 1979; Jeffery and Basu 1996; National Research Council 1999; UN 1987). Women with higher levels of education universally have fewer children, because they marry later and less often, have greater autonomy in reproductive decision-making, have more knowledge and better access to contraception, use contraception more effectively, and are more motivated to limit childbearing because of its higher opportunity costs (Bongaarts 2010; Jejeebhoy 1995). The effect of education on fertility is particularly strong in countries that still have relatively high overall fertility levels (Skirbekk 2008). Therefore, in countries in early stage of demographic transition, small increases in female literacy rate can generate a substantial decrease in birth rates and spark demographic change. Demographic modeling exercises indicate that the global population outlook depends greatly on further progress in education, particularly of young women (Lutz and KC 2011).

### Population and economic growth

#### Population affects economic growth

The impact of population growth on economic growth is very controversial among scholars. On the one hand, Malthusians and Neo-Malthusians maintain that population growth causes physical capital dilution and leads to lower productivity and slow economic growth. In areas where natural resources are managed as common property, greater population will lead to pressures on and ultimately degradation of the natural resource base. Increased scarcity of natural resources leads to greater demand for children (Nerlove 1991), as the marginal value of children’s time relative to adults’ time rises. If the substitution effect outweighs income effect, the worsening environmental conditions will increase demand for children in spite of the fact that an additional child worsens resource scarcity for all other families. This leads to a vicious cycle: greater population leads to worsening environment and economic condition, and worsening environment and economic condition leads to more rapid population growth (Dasgupta 1995).

On the other hand, many authors argue that population growth promotes economic growth through a positive impact on the state of knowledge and progress in technology (Kuznets 1973; Simon 1981, 1986). The Malthusian trap could be overcome by sufficient investment in human capital, making up for relative scarcity of physical capital (Becker et al. 1990). The history of the world has shown that periods of low population growth have been periods of low economic growth and that high rates of economic growth have occurred when population growth is also high (Johnson 1999), because while productivity is determined by land areas, technology and human capital, technology (productivity) is endogenously determined by population growth (Kremer 1993). Population pressure triggers technology improvement in agriculture, changes in location of population, and prevents undesirable pressure on natural resources (Boserup 1981; Clark 1967). Industrialization mostly occurs in regions of high population density (Malmberg and Lindh 2002). Population growth also causes increasing demand, which attracts investment (Ogliett 2007); a large and dense population increases returns to investment in human capital and research, accelerates technological innovation, and ignites economic growth (Voisenat 1987).

Other authors find that the positive contribution of population growth occurs only in the long run (Hussain et al. 2009; Zanker 2004). There is no relationship between population growth and economic development in the short run, because the positive productivity effect and the negative capital dilution effect offset each other (Mankiw et al. 1992). Moreover, the impact of population growth on the long-term economic growth can be explained by research intensity and education attainment (Jones 2002). Technological change only occurs in countries that have enough human capital and advanced institutions (Kremer 1993). It is not population size or growth that matters for technological change, but rather research intensity and efforts (Mankiw et al. 1992). Moreover, the fruits of technological progress cannot be captured exclusively by the country that produces them, but somehow get diffused to the rest of the world (Jones 2002). A study of developing countries during 1965–1985 indicates that developing countries were able to shift labor from low productivity agriculture to high productivity industry and the service sector despite rapid population growth; it is the timing and components of population growth that are important elements in the process of economic development (Bloom and Freeman 1988). At any given population growth rate, income growth is related to the time path of population growth; population growth due to high birth and death rates is associated with slower income growth than population growth due to relatively low birth and death rates.

#### Economic growth affects population

Studies of demographic history indicate that socioeconomic transition has been accompanied by demographic transition (Notestein 1945, 1953). Modernization and industrialization changed the characteristics of high fertility and mortality, and the decline in mortality preceded the decline in fertility and brought about a period of rapid population growth (Coale 1973). While economic development has always been negatively associated with mortality and contributes positively to population growth, Clark (2005) finds there are different behaviors in the link between income and fertility across different stages of development, which makes constructing a link between population growth (which depends more on fertility) and income challenging.

In the agrarian economies, most of the studies document a positive relationship between income and fertility (Hagen 1959; Simon 1986; Skirbekk 2008), mainly because high income and wealthy families can afford to have more children (Galor and Moav 2002). This relationship becomes negative after industrialization (Jones et al. 2008; Jones and Tertilt 2008), determined by factors including rising income and growth of factory employment (Notestein 1953). Becker (1960) argues rich people have fewer children because they are more expensive for parents with higher wages in terms of opportunity costs. Rising benefits from investment in human capital lead to reallocation of expenditure from numbers to quality (Galor and Moav 2002). Increasing female earnings also significantly contributes to fertility decline. A study of 83 countries in 1995 finds a 10 % increase in female labor force participation is associated with 0.45 fewer children per women (Feyrer et al. 2008).

However, Clark (2005) indicates that in modern high-income but low-fertility societies, there seems to be no association between income and fertility. The negative relationship between economic growth and fertility is mainly a statistical fluke. Once the missing variables are controlled for, fertility and income are actually positively related. For instance, the income level of men is indeed weakly positively correlated with fertility once correctly controlling for female income (Jones et al. 2008). Even the negative relationship between female labor force participation and fertility has changed over time, shifting from negative in the 1970s to zero in the 1980s–1990s, and to an upward-slope by 2000 (Mira and Ahn 2002). It suggests a U-shaped relationship, with the turning point at 50–60 % female labor force participation. This variation is due to changes in the institutional context, such as changes in childcare availability and attitudes toward working mothers that might have reduced the incompatibility between childrearing and the employment of women (Engelhardt et al. 2004). Ranjan (1999) explains the sharp decline in birth rates in the former Soviet Republics and Eastern European countries and suggests that it may be optimal for individuals to postpone childbearing during times of increased income uncertainty. Therefore, economic growth and income stability may contribute to higher fertility in very low-fertility countries.

### Education and economic growth

#### Education affects economic growth

Most existing studies suggests that the impact of investment in education on national economic growth is positive (Hannum and Buchmann 2003a; Hanushek and Wossmann 2007; Temple 2001; Wilson and Briscoe 2004). At the individual level, people’s earning can be seen as a function of education and other factors such as age and experience. Mincer (1974) finds that an extra year of education raises the earnings by about 7 % for US white males. At national level, education is needed for countries to make good use of available technology. Stevens and Weale (2003) reveals that a 1 % increase in primary school enrollment rate raises GDP by 0.35 %, and the rates of return from investment in education are found in the range of 6–12 %.

However, studies also suggest that marginal economic returns decrease with the amount of education received by individuals and with the income level of the country concerned. One extra year of education raises labor income by about 10 % in a developing country, but by only 6.5 % in an advanced economy (Psacharopoulos 1994). Hence, there may be a possibility of over-education in countries with very high education levels. Moreover, the positive impact of educational attainment on economic growth needs a long time to ripen (Lutz et al. 2008; Temple 2001). Stevens and Weale (2003) find that higher levels of GDP growth are associated with higher levels of primary enrollment some 30 year earlier. As a result, macroeconomic analysis shows that changes in educational attainment are largely unrelated to the current economic growth rate (Benhabib and Spiegel 1994). Prichett (2001) even finds primary enrollment negatively related to income growth, because in many poor countries the provision of basic education is a great burden on society. Voisenat (1987) indicates that low levels of school attendance seem to be a burden on the economy rather than a booster of productivity, and only when the threshold of about 4 years of schooling is reached does the effect of education on productivity become positive.

Lutz et al. (2008) indicate that the ambiguity in the empirical evidence relating education to economic growth is largely due to the deficiencies in the series of education data. Using the new educational attainment data by age groups, their study shows significantly positive education impacts on economic growth for some age and education groups. Other authors, on the other hand, argue that it is difficult to find a clear causal relation between education and economic growth, because it is cognitive skills rather than mere school attainment that is powerfully related to individual earnings, the distribution of income, and to national economic growth (Hanushek and Wossmann 2007); education enhances but does not ensure an individual’s economic security, since it does not necessarily narrow social inequality or enhance democratization (Hannum and Buchmann 2003b).

#### Economic growth affects education

Many authors argue that growing income is the most important driver of mass education (UNDP 2003; World Bank 2003). Lack of income is generally perceived as the most significant barrier to educational expansion. In low-income countries, households derive a significant portion of their income from child labor and are therefore less motivated to send children to school (Fuller and Rubinson 1992; Weiner 1991). Moreover, the poorer the country, the more difficult to increase public expenditure on education and improve education quality (Clemens 2004), and the more reluctant are the parents to send children to school because of the perceived low economic returns to education (Probe Team 1999). A higher aggregate income level allows states to invest more in education and improving education quality, which significantly contributes to education expansion. As family income increases, the ability and willingness of citizens to temporarily forgo income to continue their education also increases. This explains why higher national income level leads to increased societal demand for education (UNDP 2003; World Bank 2003).

However, Easterlin (1981) indicates that the spread of formal education in Northwestern Europe, North America, and some developing countries seems to have preceded the beginning of modern economic growth. Studies of the cross-section and cross-time statistical analyses do not find any positive effects of income on average schooling years of the population (Easterly 1999). An event-history analysis, based on newly available enrollment data for over 120 countries for the period 1870–1980, reveals that expansion of mass education is endemic in the system, organized politically as nation-states or candidate states. It appeared at a steady rate before the 1940s but sharply increased after 1950 (Meyer et al. 1992).

### Summary

**Fig. 2:**
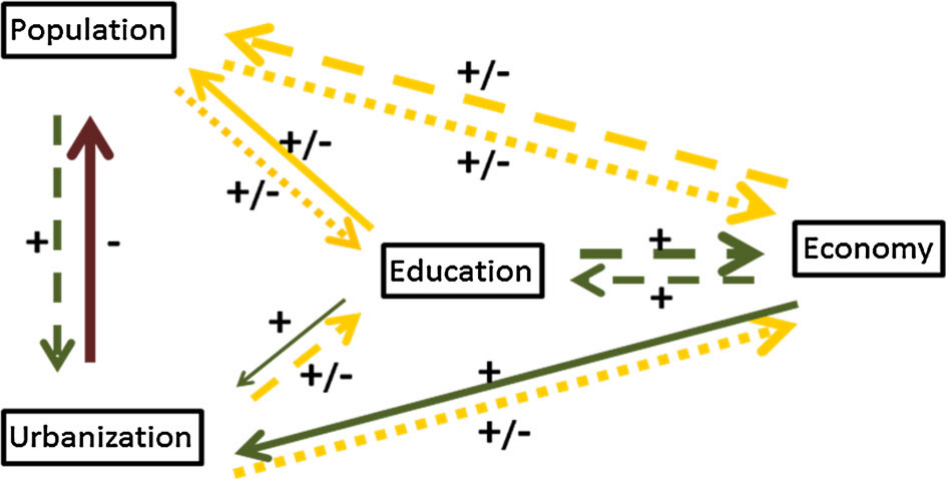
Interactions of changes in population, urbanization, education, and economy in the literature. *Notes* 1. “+” denotes dominantly positive; “−” dominantly negative; “±” unclear or change in various stages of development and urbanization. 2. *Solid line* high agreement; *dashed line* medium agreement; *dotted line* low agreement; 3. *Thick line* robust evidence; *normal* medium evidence; *thin line* limited evidence

To summarize the findings from the literature review, Fig. 2 plots the direct and indirect causal relationships between each of the four factors, accounting for the direction of the effects (positive or negative), degree of consensus among the literature (high, medium or low agreement), and the evidence from the amount of studies (robust, medium, or limited evidence). Moreover, based on the causal relations established by the literature, we are able to derive the signs of dominant effects between each pair of the four factors for regions at different development levels and for the whole world (Table 2).

**Table 2:** Sign of correlations between changes in population, urbanization, education, and economic growth based on findings from the literature

	Urb*GDP	Urb*Pop	Urb*Edu	Pop*GDP	Pop*Edu	Edu*GDP
World	+	+/−	+	+/−	+/−	+
More developed	+	+/−	+	+/−	+/−	+
Medium developed	+/−	+	+	+/−	+/−	+
Low developed	+/−	−	+/−	−	−	+

Same as Table 1

Comparing the signs of correlations between these factors suggested by the literature (in Table 2) with those implied by assumptions in the SSPs projections (in Table 1), we find that they match reasonably well with each other. More specifically, the correlations between changes in education and per capita GDP (Edu*GDP) are all positive for regions at various development levels in both Tables 1 and 2; the relationships between urbanization and GDP (Urb*GDP) and education (Urb*Edu) are also dominantly positive and shows a consistent pattern in both tables. More importantly, the signs of relationships implied by the assumptions under each SSP projection can all find confirmation in the literature, except for the relationships Pop*GDP, Pop*Urb, and Pop*Edu for low-income countries under SSP2. This exception may not be a serious inconsistency problem, because the same medium assumptions for different factors follow in fact different paths in different development stages. We return to this point in the next section.

## Examining the interactions in the projection results

The previous section showed that the interactions implied by the assumptions in the SSP projections are reasonably consistent with findings in the existing research literature. This section examines whether these same implied relationships are consistently represented in the projection results produced by the different modeling teams. Data on the SSP projection results are obtained from the SSP database. For GDP projections, we use the projection of the OECD modeling group since it is considered the “marker” of GDP projections for the SSPs. We derive the aggregate statistics for regions by income and for the world based on the national level projections and weighing the projected population of countries in each region.

**Fig. 3:**
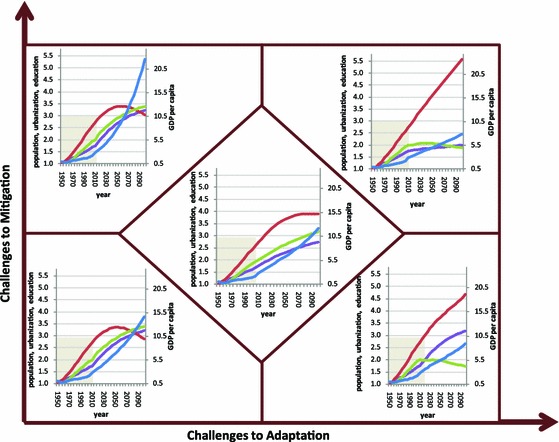
Historical and projected relative changes in global population (*red*), urbanization (*purple*), education (*green*), and economic growth (*blue*) under SSPs (1950 = 1). *Notes* the historical data for the period of 1950–2010 (*shaded areas*) on population is from *World Population Prospects 2010 Revision* (United Nations 2011), urbanization is from *World Urbanization Prospects 2009 Revision* (United Nations 2010), education (mean year of schooling of population aged 15+) is from IIASA (Lutz et al. 2007), and per capita GDP (PPP 2005$) is from Penn World Table (Heston et al. 2011). The data for the future (2010–2100) is derived from SSPs database (https://secure.iiasa.ac.at/web-apps/ene/SspDb/dsd?Action=htmlpage&page=welcome). Because global per capita GDP declined during the 1950s and the early 1960s, the scale of relative changes in per capita GDP (*the right vertical axis* of each chart) starts from 0.5 (Color figure online)

Figure 3 shows the relative changes (1950 = 1) in global population, urbanization, education (mean year of schooling of population aged 15+), and per capita GDP under each SSP. At the global level, the projection results on the four factors generally match the changes expected from the qualitative narratives and quantitative assumptions under each SSP. Along the diagonal of the chart, low population growth but high urbanization, education and GDP growth are observed under SSP1; high population growth but low urbanization, education, and GDP growth occur under SSP3; and medium growth in all the four factors are found under SSP2. On the off-diagonal matrix spaces, SSP4 has medium (close to SSP2) population growth, fast urbanization, very low education, and low (close to SSP3) GDP growth, while SSP5 has low (close to SSP1) population growth, fast urbanization and high education growth, and very high per capita GDP growth. We calculate the bivariate correlation coefficients between projected changes in each pair from the four factors at the global and regional level, and summarize the results in Table 3.

**Table 3:** Bivariate correlation coefficients of the projected changes in population, urbanization, education, and per capita GDP for regions by income and for the world under SSPs

SSP	Region	Urb*GDP	Urb*Pop	Urb*Edu	Pop*GDP	Pop*Edu	Edu*GDP
SSP1	World	0.544	0.588	0.601	0.451	0.558	0.299
	High income	0.164	−	0.416	–	0.570	–
	Medium income	0.616	***0.387***	0.543	***0.414***	***0.659***	0.413
	Low income	0.306	***0.662***	0.573	***0.339***	***0.540***	–
SSP2	World	0.552	0.577	0.604	0.477	0.653	0.448
	High income	0.260	–	0.345	–	0.559	0.143
	Medium income	0.595	0.270	0.457	0.309	0.615	0.384
	Low income	0.210	0.568	0.499	0.216	0.536	0.136
SSP3	World	0.422	0.486	0.134	0.398	–	–
	High income	0.190	0.351	0.316	0.210	0.368	0.236
	Medium income	0.443	***0.121***	0.224	***0.132***	***0.229***	0.170
	Low income	–	***0.294***	0.125	–	–	0.160
SSP4	World	0.480	0.537	−0.157	0.283	−0.136	–
	High income	0.223	–	0.255	–	0.282	0.105
	Medium income	***0.637***	***0.271***	–	0.147	–	–
	Low income	***0.178***	0.445	−0.205	–	−0.177	0.119
SSP5	World	0.526	0.497	0.601	0.302	0.494	0.275
	High income	–	**−** ***0.156***	0.416	**−** ***0.255***	0.369	−***0.173***
	Medium income	0.589	***0.338***	0.542	***0.334***	***0.634***	0.362
	Low income	0.297	***0.650***	0.572	***0.318***	***0.532***	–

“−” non-significant; the values in bold and italic are those different from the implied relationships by the assumptions; others same as Table 1

* GDP* gross domestic product, *SSP* shared socioeconomic pathway

It shows that while the majority (82.5 %) of the signs of correlations between the factors in the projection results (Table 3) are the same as for the implied relationships by the assumptions (Table 1), some correlation coefficients (marked in bold/italic) show different signs. The differences mainly occur to the relationships between population and other factors (Pop*GDP, Pop*Edu, Urb*Pop), particularly for low- and medium-income regions under SSP1, SSP3, and SSP5. One possible reason for the differences is due to the fact that the definition of income regions used in the population and education projections is slightly different from that used in the GDP and urbanization projections. While the latter is based solely on income levels, the former considers the differences in fertility. Even though fertility levels are often highly correlated with income levels, countries of the same income group may fall into different fertility groups. The difference in region definition may be partly responsible for the inconsistency in the relationships between population and GDP growth and the relationships between population and urbanization. However, it cannot explain the inconsistency in the relationships between population and education, given that the population and education projections use the same region definition and both are produced by the IIASA model.

Another inconsistency occurs in the relationships between urbanization and GDP growth for medium- and low-income regions under SSP4. The assumed negative correlation between urbanization and GDP for those countries under SSP4 aims to represent the situations of “rapid urbanization without growth” (Fay and Opal 1999; Oucho and Gould 1993), which occurred in some Sub-Saharan African and Latin American countries in the past. It does not appear as expected in the projection results. This may be due to the fact that these countries may be increasingly and highly urbanized in the future, and the interactions between urbanization and GDP vary in different stages of urbanization and economic development. This is particularly true for the medium-income countries. The other small inconsistency occurs in the relationships between education and GDP growth under SSP1 and SSP5, which may also be due to the same reason: As human societies under SSP1 and SSP5 enter an era with advanced education and economic development levels, the interactions between education and economic growth may shift into different paradigms. This is particularly true for high-income countries. Further analysis of these interactions by stages of urbanization and economic development could help better understand the evolving patterns of changes in these factors and their interactions.

Moreover, the positive correlations of Pop*Urb, Pop*GDP, and Pop*Edu implied by the assumptions for low-income countries under SSP2, which differ from the findings in the literature, are actually consistently reflected in the projection results. It is noteworthy that the literature revealing negative relationships between these factors is based on the data from some less developed countries during certain periods in the past. And, the countries included in the SSPs low-income regions already display some variations in development levels at the present time, and we may anticipate larger differences in the speed and timing of demographic and economic transitions in future decades. The negative correlations suggested by the literature for Pop*Urb, Pop*GDP, and Pop*Edu may occur for some low-income countries during some periods of time, but we may expect they will not occur for the whole income group or for the whole projection period.

## Conclusions

Our research shows that the inclusion of a comprehensive set of demographic and economic factors in the SSPs is well designed, and the resulting scenarios convey coherent stories about future development pathways. The implied interactions by the combination of group assumptions on population, urbanization, education and economic growth under SSPs are generally consistent with findings from the literature, and appropriately reflected in the projection results produced by individual modeling teams.

This analysis also reveals some issues of inconsistency existing in a few implied interactions by the demographic assumptions in the SSPs for some income country regions, which differ either from those suggested by the literature or from those appearing in the projection results. Some inconsistency problems can be attributed to the differences in definition of the SSP regions used in different SSPs projections. Those problems can be avoided relatively easily in the future works. However, to avoid some other inconsistency issues, we need to improve our understanding about the evolving relationships between these demographic and economic factors in human history and their possible changes in the future. For instance, we need to explore the patterns of these evolving interactions in different stages of economic development and urbanization through analyzing the historical statistics and comparing with the results in the SSPs projections. That research is beyond the scope of this paper and will be reported in another article.

Moreover, the analysis in this paper is based only on the first set of global basic SSPs. The climate change research communities are currently developing extended SSPs for research at the national and local levels. The assumptions in the extended SSPs based on national and local circumstances can also produce more consistent results and contribute to better understandings of the variations in the interactions between the demographic and economic determinants in different stages of socioeconomic development and demographic transitions. In fact, some relationships implied by the combinations of assumptions that have inconsistency issues at the global or region levels could prove to be reasonable in the analysis at local levels.

## Footnotes

1 In this analysis, unless otherwise specified, the test of the assumptions and their implied relationships are all based on the changes (annual growth rates), not the levels, of the demographic and economic elements.
